# Congenital trans-sellar trans-sphenoidal encephalocele: a systematic review of diagnosis, treatment, and prognosis

**DOI:** 10.1007/s00405-023-08355-5

**Published:** 2024-01-08

**Authors:** Zheng Jiang, Deming Yang, Mailudan Ainiwaer, Qiong Li, Wei Mo, Feng Liu

**Affiliations:** 1 Department of Otolaryngology, Head and Neck Surgery, West China Hospital, Sichuan University, Chengdu, 610041 Sichuan Province China; 2 Department of Otolaryngology, Zhaotong Second People’s Hospital, Zhaotong, 657099 China; 3 Department of Otolaryngology, The People’s Hospital of Jianyang City, Chengdu, 641499 China

**Keywords:** Encephalocele, Congenital, Trans-sellar, Trans-sphenoidal

## Abstract

**Purpose:**

Clinical presentations encompass respiratory, feeding issues, nasopharyngeal mass, meningitis, CSF leakage, craniofacial anomalies, and endocrine problems. Surgery is the primary treatment, transitioning from frontal craniotomy to endoscopic methods, offering improved outcomes. Yet, more studies are needed. A comprehensive review on trans-sellar trans-sphenoidal encephalocele (TSTSE) is missing. Our study aims to fill this gap, offering a comprehensive perspective for physicians.

**Methods:**

This review adhered to the PRISMA guideline. Eligible studies focused on human subjects, specifically trans-sellar encephaloceles, and provided comprehensive treatment details. English language articles published up to April 11th, 2023, were considered. Two trained researchers conducted article screening using consistent criteria. Data extraction covered various aspects, including clinical presentation, surgical methods, and outcomes, with results presented descriptively in two tables. Due to the rarity of this congenital anomaly, meta-analysis and publication bias assessment were not feasible. Data extraction was independently conducted by two reviewers, with subsequent cross-verification.

**Results:**

A total of 36 patients were identified from 14 studies, the most frequently observed clinical presentation was dyspnea (41.67%) and the most frequently observed accompanying anomaly was cleft lip/palate (55.56%). CT and MRI were adopted in nearly all the cases, and trans-nasal approach was the most often used surgical approach (57.14%) with the ‘soft material combination’ the most commonly used method for cranial base repairment (35.71%). A total of two deaths occurred and diabetes insipidus was the most common perioperative complication which occurred in six surgery patients (21.43%).

**Conclusion:**

TSTSE predominantly affects males and presents with dyspnea, visual deficits, pituitary insufficiency, and cranial base-related symptoms. Early diagnosis is critical, with advanced imaging playing a key role. Endocrine assessment is vital for hormone management. Surgery offers symptom relief but entails risks, including reported fatalities and complications. The choice between surgery and conservative management requires careful deliberation. The trans-nasal approach is favored for its reduced trauma, yet further research is necessary to validate this preference.

## Introduction

Pediatric encephalocele, a congenital malformation characterized by the protrusion of intracranial structures through a cranial defect, presents an overall incidence of around 0.8–3.0 per 10,000 live births [1, 2]. Among its subtypes, basal encephaloceles, accounting for a mere 1.5% of all encephaloceles, exhibit a remarkable rarity with an estimated incidence of 1 in 35,000 live births [3]. Basal encephaloceles have conventionally been categorized into five distinct anatomical types: spheno-ethmoidal, trans-sphenoidal, trans-sphenoidal trans-sellar, spheno-orbital, trans-ethmoidal, and spheno-maxillary [4]. With the trans-sphenoidal trans-sellar being the rarest among all [3, 5].

The precise etiology of trans-sphenoidal encephaloceles (TSE) remains elusive. However, it is widely hypothesized to arise from an embryological defect in the skull base. Among the proposed mechanisms, the persistence of the craniopharyngeal canal (CPC) is currently the most widely accepted theory explaining the pathogenesis of TSE [5–7]. It is postulated that an early intrauterine insult occurring during the critical period of pituitary development may give rise to cascading effects on underdeveloped tissues such as the optic chiasm and osseous sphenoid elements.

Clinical presentations include regional symptoms like respiratory difficulties, feeding difficulties, pulsatile nasopharyngeal mass, recurrent episodes of meningitis, and cerebrospinal fluid (CSF) rhinorrhea; associated craniofacial anomalies like cleft lip and palate, ocular anomaly, craniosynostosis, hypertelorism; endocrinal presentations like hypothyroidism, hypopituitary-related growth retardation, diabetes insipidus, and so on [5, 8, 9].

Current treatment options are primarily surgeries. Frontal craniotomy was the most commonly adopted procedure in the past decades [3], with the endoscopic methods like trans-palatal or trans-nasal being more popular in recent years [8, 10, 11]. Though several studies have concluded that endoscopic approaches have generally improved closure rates with decreased morbidities [2], more studies are required to consolidate the clinical evidence.

To date, a comprehensive review focusing on the diagnosis, treatment, and prognosis of trans-sellar trans-sphenoidal encephalocele (TSTSE) is notably lacking. Therefore, the objective of this study is to systematically investigate the entire treatment trajectory associated with TSTSE, thereby providing physicians with a comprehensive perspective on the clinical management of this condition. Through a meticulous examination of available literature and clinical experiences, our research endeavors to fill the current knowledge gap and contribute to improved understanding and enhanced care for patients with TSTSE.

## Methods

This review was in accordance with the PRISMA guideline [12].

### Search strategy

Literature searches were conducted in PubMed, MEDLINE, and Google Scholar. Relevant peer-reviewed articles were included based on titles and abstracts. Keywords included encephalocele, meningoencephalocele, meningocele, trans-sellar/trans-salar, and trans-sphenoidal. In addition, we searched reference lists of published studies, meta-analyses, and review articles for more eligible articles. The last search took place on April 11, 2023. Article screening was performed by two trained research staff using the same formula.

### Study eligibility

Included studies should meet the following criteria: (1) human objects only; (2) the pattern of encephalocele should be specified as trans-sellar; (3) studies should include detailed treatment course of the patients including the drug, surgical procedures as well as postoperative outcome; (4) the study was an original research report; and (5) articles written in English.

### Data extraction

All data were extracted for the following variables: (1) article title, (2) 1st author, (3) study design, (4) age, (5) gender, (6) clinical presentation, (7) congenital anomalies, (8) diagnostic tests, (9) abnormal lab results, (10) type of surgical approach, (11) materials used in cranial base repairment, (12) perioperative complications, and (13) treatment outcomes. Meta-analysis or publication bias assessment could not be conducted because this is a rare congenital anomaly, and all the studies are either case reports or case series. Data extraction was performed by two reviewers independently and cross-checked afterward.

### Result synthesis

The data were tabulated into two tables: Table 1 consists of basic demographic and clinical presentation data. Table 2 summarizes key information of studies, including the surgical approach, materials used to repair cranial base, perioperative complications, and outcomes, wherein the main outcomes were summarized into brief sentences. All the data were presented in descriptive pattern.

**Table 1:** Basic demographics, clinical presentation, and diagnostic tests

Author	Patient no.	Sex/age	Clinical presentation	Anomalies	Diagnostic tests	Abnormal lab results
Bhaisora [3]	1	18mo/M	Compressible cystic swelling with cough impulse	Right microphthalmia, complete corneal opacity, midline cleft palate, cleft lip	CT, MRI	Hypothyroidism
Raman Sharma [16]	2	5mo/M	Nasal obstruction, a mass in the mouth	/	CT, MRI	Not specified
	3	14yo/M	Chronic nasal obstruction	/	CT, MRI	Not specified
Spacca [17]	4	14mo/M	Dyspnea	Ogival palate, coloboma	CT, MRI	LH-FSH, TSH deficit
	5	18yo/M	Visual loss	/	CT, MRI	Panhypopituitarism
	6	3yo/F	Dyspnea, strabismus, exotropia of eyes	Callosal agenesis	CT, MRI	GH deficit
	7	14yo/M	Strabismus, dyspnea	Callosal agenesis	CT, MRI	Panhypopituitarism
	8	5yo/M	Dyspnea	/	CT, MRI	GH deficit, hypopituitarism
	9	9yo/M	Recurrent meningitis, diabetes insipidus	/	CT, MRI	Panhypopituitarism
Yang [2]	10	3yo/M	Dyspnea, strabismus, developmental delay	/	CT, MRI	Testosterone, fT3, cortisol, TSH deficit
	11	5yo/F	Obesity, mammary gland growth, galactorrhea	/	CT, MRI	Elevated PRL
	12	2yo/M	Developmental delay, visual deficit, dyspnea, CSF leak	Cleft palate	CT, MRI	Cortisol, testosterone deficit
	13	17yo/M	CSF leak, visual deficit	Genital abnormalities	CT, MRI	Testosterone deficit
	14	6yo/M	Visual deficit, dyspnea, developmental delay	Cleft palate	CT, MRI	Testosterone, fT3, cortisol, TSH deficit
	15	28yo/M	Dyspnea, polydipsia and polyuria, visual deficit, thermoregulation disorder	Cleft palate, genital abnormalities	CT, MRI	Testosterone, cortisol, TSH deficit
Zeinalizadeh [10]	16	18mo/F	Nasal airway obstruction	Cleft lip, hypertelorism	CT, MRI	Normal
	17	24mo/M	Failure to thrive, nasal obstruction, polydipsia, and polyuria	Cleft lip	CT, MRI	Not specified
	18	8mo/F	Upper airway obstruction	/	CT, MRI	Normal
Mylanus [14]	19	7mo/M	Failed to thrive, inspiratory stridor, progressive snoring, and serious episodic oxygen desaturations during sleep	Asymmetrical face	CT, MRI	Not specified
Franco [11]	20–25	3mo–4yo/all M	Nystagmus, strabismus	Cleft lip, cleft palate, hypertelorism, lingual tumor-like masses	MRI	Not specified
Rathore [8]	26	5mo/M	Mass in mouth with feeding and respiratory difficulty	Cleft palate	CT, MRI	Normal
	27	14yo/M	Chronic nasal obstruction	Cleft lip and hypertelorism	CT, MRI	Normal
	28	4yo/M	Spontaneous CSF rhinorrhea with recurrent meningitis	Cleft lip and hypertelorism	CT, MRI	Normal
	29	16mo/M	Mass in the mouth with respiratory and feeding difficulty	Cleft lip and palate with hypertelorism	CT, MRI	Normal
Formica [18]	30	8mo/F	Dyspnea, steady stridor-like breathing, impairment of visual acuity	Hypertelorism, cleft palate and lip	CT, MRI	Normal
Kumar [24]	31	30d/M	Difficulty breathing and feeding	/	CT, MRI	Not specified
Steven [13]	32	8d/M	Mouth-breathing, subcostal and suprasternal recession	/	CT, MRI	Not specified
Pradhan [25]	33	Neonate/F	/	Cleft lip, cleft palate, broad nasal alar	CT, MRI	Normal
Saito [19]	34	36yo/M	Nasal obstruction	Coloboma, cleft palate	CT, MRI	Mild panhypopituitarism
Zoghlami [15]	35	3.5yo/M	Recurrent meningitis	/	CT, MRI	Not specified
	36	36yo/M	Recurrent meningitis, CSF leak	/	CT, MRI	Not specified

**Table 2:** Data associated with surgical intervention

Author	Patient no.	Surgical approach	Materials used in strengthening the cranial base	Perioperative complications?	Treatment outcomes
Bhaisora [3]	1	Transcranial	Fascia lata overlay graft, reinforced with small bone piece and fibrin glue	Uneventful	Discharged on the 7th postoperative day, no CSF leak at 6-month follow-up
Raman Sharma [16]	2	Trans-palatal	Split rib graft fixed with mini-plate and screws	CSF leak on the 10th postoperative day and developed fulminant meningitis	Death
	3	Trans-nasal	Split rib graft fixed with mini-plate and screws	Uneventful	Good postoperative recovery and satisfactory follow-up for 2 years
Spacca [17]	4	Transcranial, trans-palatal	Multiple levels of human deantigenic bone or demineralized human bone matrix and sealed with oxidated regenerated cellulose and fibrin glue	Not specified	Gonadic improvement but relapse of the encephalocele and reoperation after 11 years. After the reoperation, palatal dehiscence completely recovered; no hormone replacement therapy needed
	5	Trans-palatal	Same as case 4	Not specified	Improvement in visual and hormone functions on 8-year follow-up
	6	Trans-nasal	Same as case 4	Not specified	Require GH substitutive therapy; no visual impairment on 8-year follow-up
	7	Trans-nasal	Same as case 4	Not specified	No more dyspnea, no hormone replacement therapy needed, no visual impairment on 5-year follow-up
	8	Conservative	/	Not specified	Panhypopituitarism, obstructive dyspnea on 7-year follow-up
	9	Trans-palatal	Same as case 4	Not specified	Palatal dehiscence completely recovered, normal visual function on 2-year follow-up
Yang [2]	10	Trans-nasal	Muscle and fat, and used iodoform gauze to sustain the new sellar floor	Fever, severe diabetes insipidus, electrolyte disturbances	No dyspnea, visual function and hormone improved on 6.5-year follow-up
	11	Trans-nasal	Same as case 10	Transient diabetes insipidus, electrolyte disturbances	Normal hormone on 9-year follow-up
	12	Trans-nasal	Same as case 10	Transient diabetes insipidus, electrolyte disturbances	No dyspnea, visual impairment or CSF leak on 6-year follow-up
	13	Trans-nasal	Same as case 10	Transient diabetes insipidus, electrolyte disturbances	No CSF leak, visual function and hormone improved on 4-year follow-up
	14	Trans-nasal	Same as case 10	Severe transient diabetes insipidus, electrolyte disturbances	Normal visual function, no hormone replacement therapy needed on 1-year follow-up
	15	Conservative	/	Uneventful	Obstructive dyspnea, hormone replacement therapy needed on 9-year follow-up
Zeinalizadeh [10]	16	Trans-nasal	Free fascia lata graft, titanium mesh plate was affixed to the clivus with screws, then cover with pedicle nasoseptal flap	Uneventful	Had no further respiratory distress, CSF leak, or endocrinological changes, no recurrence of sac on 2-year follow-up
	17	Trans-nasal	Same as case 16	Uneventful	At 1-year follow-up the patient had regained the normal weight for his age, and had no CSF leak or any other complications
	18	Trans-nasal	Same as case 16	Transient episode of diabetes insipidus, pneumonia	Complete resolution of the meningoencephalocele on 6-month follow-up
Mylanus [14]	19	Transcranial + trans-palatal	Free galea periosteum flap and fibrin glue	Right abducens paresis	Complete disappearance of the abducens paresis. No more respiratory problems. The patient has resumed eating and has gained weight according to his age, and his cognitive and motor development is normal
Franco [11]	20–25	One trans-nasal; Five conservative	Not specified	Uneventful	Not reported
Rathore [8]	26	Trans-palatal	Nasopharyngeal mucosal flap	CSF rhinorrhea on tenth postoperative day; Expired on fourteen postoperative days due to fulminant Kleibseilla pneumonia	Death
	27	Trans-nasal	Split rib graft	Not specified	At two-year follow-up, patient was symptomatic
	28	Trans-nasal	Septal cartilage, tensor fascia lata graft and tissue glue	Uneventful	Asymptomatic at three-month follow-up
	29	Trans-palatal	Fascia lata, titanium mesh and tissue glue	Hyponatremia on postoperative day ninth	Not reported
Formica [18]	30	Trans-palatal	Posterior parietal bone graft	Uneventful	Not reported
Kumar [24]	31	Trans-palatal	Not specified	Not specified	Not reported
Steven [13]	32	Trans-palatal	Bone paté, periosteum, post-auricular fascia and fibrin glue	Uneventful	No evidence of sac recurrence
Pradhan [25]	33	Conservative	/	/	Not reported
Saito [19]	34	Trans-nasal	Septal cartilage, pedicled vascularized nasoseptal flap, oxidized cellulose with fibrin glue	Uneventful	The patient felt improvement of visual impairment. At 1-year follow-up, MRI showed no recurrence
Zoghlami [15]	35	Transcranial	Muscle, fibrin sealant	Right parietal extradural hematoma	Reoperated twice, and the meningoencephalocele persisted on 9-year follow-up
	36	Trans-nasal	Fat, fibrin sealant	Meningitis, right trigeminal nerve palsy, facial nerve palsy	Reoperated twice, the patient had permanent right peripheral facial nerve and trigeminal nerve palsy. No recurrence of encephalocele on 18-month follow-up

## Results

**Fig. 1:**
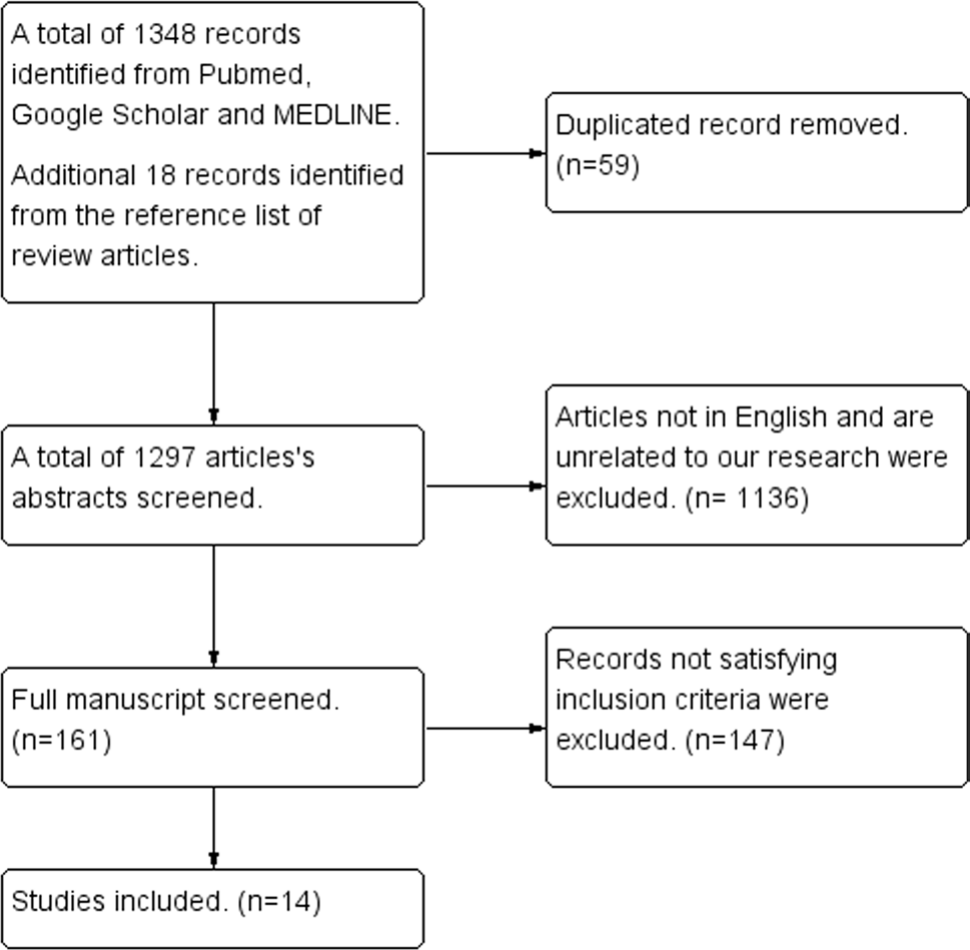
The flow diagram of the review

A total of 14 studies met the inclusion criteria and were included in this review (Fig. 1).

### Basic demographics of the patients

A total of 36 patients were identified from 14 studies. Among those patients, there was a male predominance. Out of the 36 patients, 30 were males (83.33%) and 6 were females (16.66%), the average age of documented visit of the patients with encephalocele is 6.48 years old (SD = 9.72). The mean age for surgically treatment patient was 7.02 years old (SD = 9.87). The youngest surgically treated patient was reported by Steven et al. with an age of 8 days [13].

### Clinical presentation and congenital anomalies

**Fig. 2:**
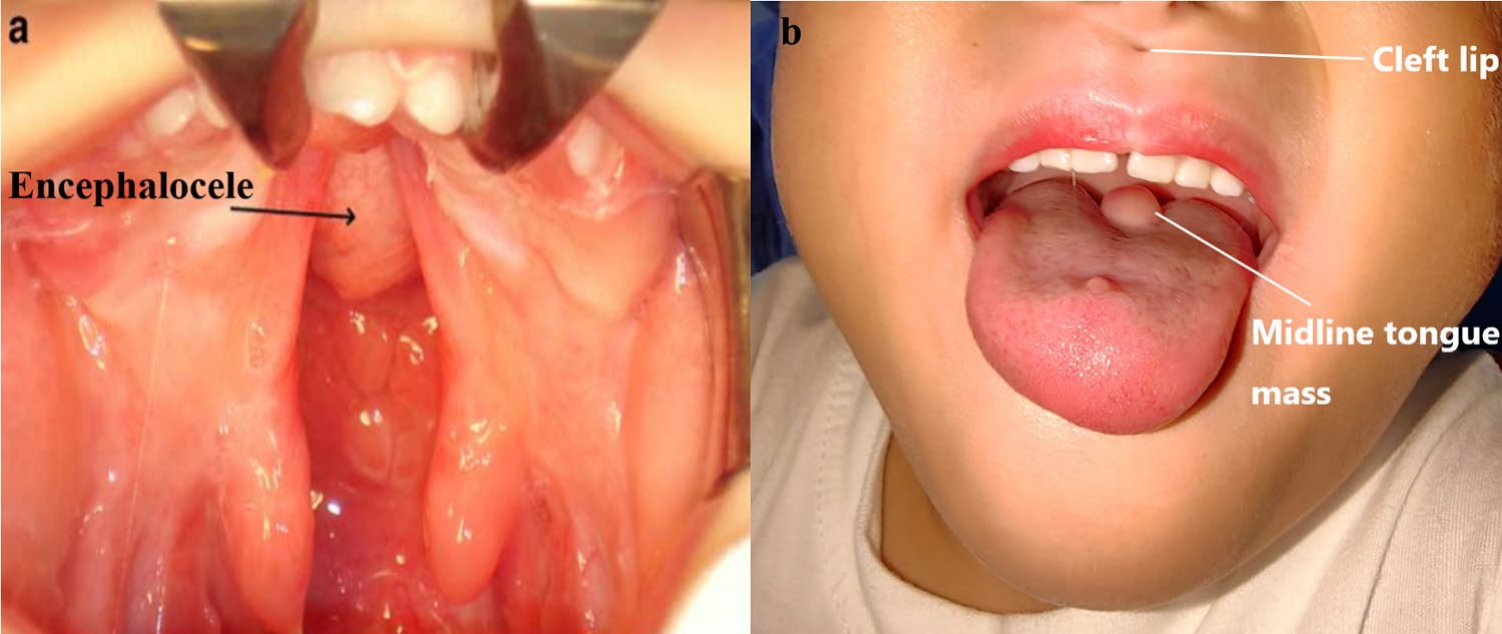
Demonstration of the related congenital anomalies. **a** Congenital cleft palate and the encephalocele; **b** Cleft lip and midline tongue mass. **a** was reproduced from Franco et al. with permission [11]

Among the 36 patients included in this study, the most frequently observed clinical presentations were dyspnea (41.67%), visual deficits (41.67%, with 16.67% were visual defects and 25% were strabismus), pituitary insufficiency (22.22%), nasal obstruction (16.67%), recurrent meningitis (11.11%) and CSF leak (11.11%). These manifestations are believed to arise from the mass effect exerted by the TSTSE. A total of 24 patients were reported to have at least one congenital anomalies other than encephalocele (66.67%). Among which, cleft palate/lip were found to be the most commonly seen congenital anomaly accompanying TSTSE, 20 out of 36 patients were reported to have cleft lip/palate (55.56%) (Fig. 2a, b). Other commonly seen congenital anomalies include hypertelorism (19.44%), eye problems (8.33%) and genital abnormalities (5.56%). Lingual midline mass was reported only in 1 patient (Table 1).

### Diagnostic measures

**Fig. 3:**
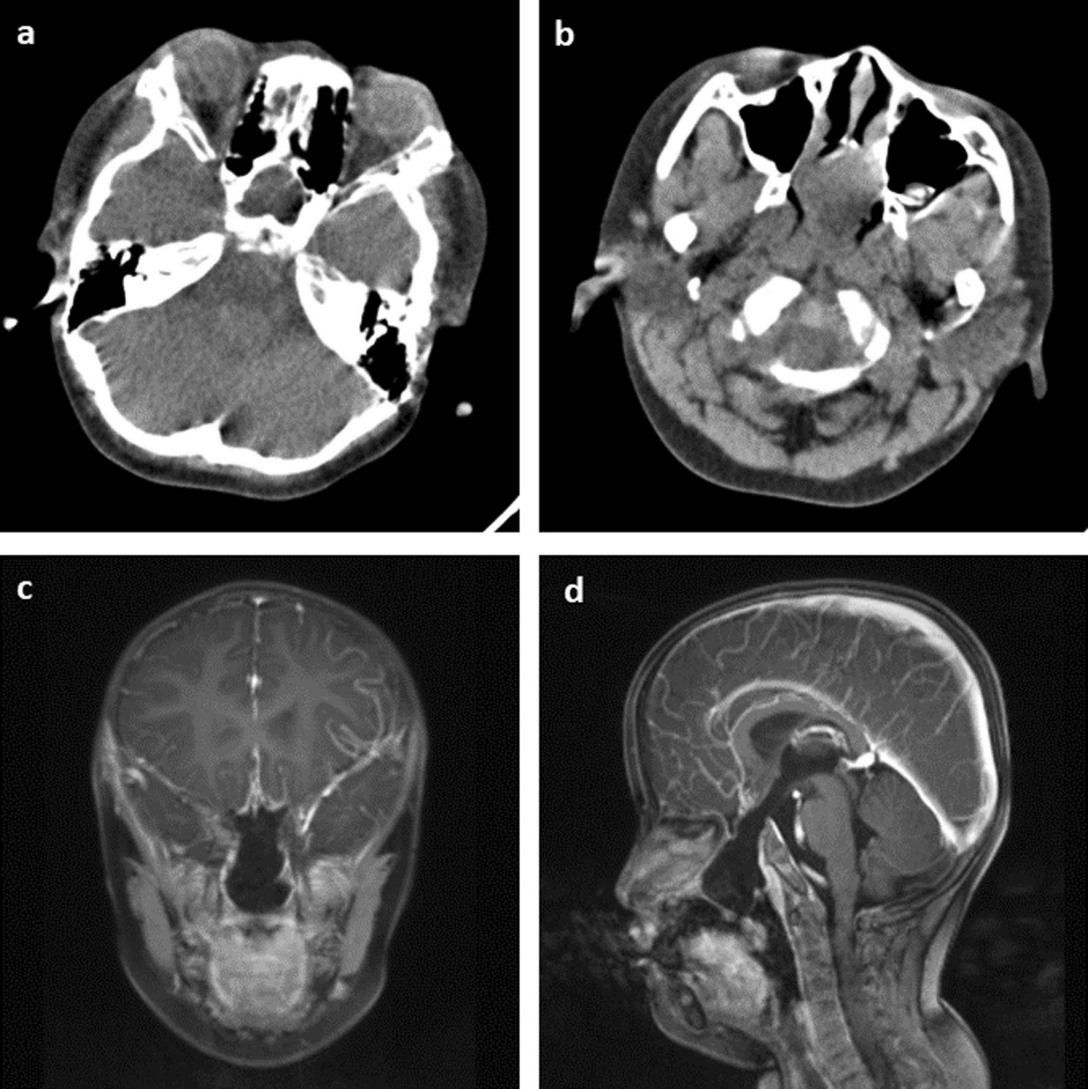
Demonstration of MRI and CT scan in trans-sellar encephalocele diagnosis. **a**, **b** CT scan showing the bony defect at the sellar region with a prominent encephalocele protruding into the nasopharynx. **c**, **d** Coronal and sagittal MRI view of the encephalocele showing that it merges with the nasal septum and obstructs the choana

Among all 14 studies, most of them prescribed MRI, CT, and hormone tests. A typical bony defect on the floor of sella and sphenoid sinus can be clearly identified in CT scan (Fig. 3a, b). Typical MRI findings include a CSF-containing sac that extends through a bony defect at the skull base which traverses the sella and sphenoid sinus before protruding into the nasal/oral cavity (Fig. 3c, d). Associated anomalies can also be identified by imaging tests. Hypothalamic–pituitary dysfunction was found in 14 out of 36 patients (38.89%), which is likely due to the dysplastic or atrophic pituitary gland and stalk resulted from TSTSE. 8 out of 36 patients (22.22%) had a normal hormone test, which can be attributed to the retaining of functional pituitary gland in the brain or as a part of the sac (Table 1).

### Surgical approach selection

Among the 36 included patients, 28 patients underwent surgical treatment. Among the surgical patients, trans-nasal approach was the most often used surgical approach (57.14%), the typical surgical procedures include (a) Harvesting nasal septal flap; (b) Encephalocele sac aspiration; (c) Separating the sac from the mucosa and reducing the sac to a normal anatomical position; (d) Reconstruction of the bony cranial base defect; (e) Reconstruction of nasal septal flap donor site.

Trans-palatal method was applied in 8 patients (28.57%), typical procedures include (a) Divide hard and soft palate to visualize the sac; (b) Dissect the sac from mucosa; (c) Cut or/and reduce the sac back to its anatomical position; (d) Strengthen the cranial base with various materials; (e) close the palatal mucosa.

Two patients underwent transcranial surgery (7.14%), and two patients underwent transcranial + trans-palatal combined surgery (7.14%). Transcranial surgery has different approaches like infratemporal fossa approach, frontotemporal trepanation or suboccipital retrosigmoid approach. Typical procedures are not essentially different from trans-nasal or trans-palatal approach, the concepts of debulking and reducing the sac as well as repairing the cranial base are similar. Some patients who underwent transcranial surgery may require additional trans-palatal surgery to remove the remaining sac locating deep in the nasopharynx (Table 2).

### Materials selected for cranial base repairment and their effect

The materials selection for cranial base repairment varied across different studies, with the majority of the surgeons chose to use more than one material in order to achieve sufficient strength of the cranial base (Table 2).

‘Soft material combination’ was applied in 10 patients and was the most commonly used regimen in cranial repairment. Yang et al. [2] used muscle and fat tissue to repair the defect and further strengthen the repairment by temporary packing with iodoform gauze. Mylanus et al. [14] used free galea periosteum flap only and sealed it up with fibrin glue. Rathore et al. [8] tried to use nasoseptal flap alone for cranial defect repairment in case 26 which did not yield ideal outcome. Steven et al. [13] used bone paté, periosteum, post-auricular fascia, and fibrin glue to repair the bony defect of a neonate. And Zoghlami et al. [15] used either simple muscle or fat with fibrin sealant in two patients which resulted in multiple reoperations. Among the 10 patients using ‘soft material combination’, 3 of them developed unfavorable postoperative complications with one of them (case 26) demonstrating severe CSF leak and eventually died from infection. The cases 35 and 36 both had 2 episodes of reoperation and severe neurological complications, with the encephalocele persisted in case 35 even after repetitive surgeries. The unfavorable outcome indicated that ‘soft material combination’, especially when only one repairing material was used, the repairment is not strong enough to sustain the cranial base.

‘Hard combination’ normally includes hard material and glue sealant, this strategy was used in 9 patients. Raman Sharma et al. [16] used split rib graft fixed with mini-plate and screws for defect repairment in two patients. Rathore et al. [8] also used split rib graft to strengthen the cranial base, the use of sealant was not reported. Spacca et al. [17] used multiple levels of human deantigenic/demineralized human bone matrix and sealed with oxidated regenerated cellulose and fibrin glue in cases 4, 5, 6, 7, and 9. Formica et al. [18] innovatively used posterior parietal bone graft; however, the perioperative and long-term follow-up course was not reported. Among the 9 patients, one patient experienced postoperative CSF leak and developed lethal meningitis. The rest 8 patients did not report any significant perioperative complications, and the long-term outcome was also preferred.

‘Hard + soft material’ combination was also a popular choice, Bhaisora et al. [3] used fascia lata and small bone pieces and sealed them up with fibrin glue. Zeinalizadeh et al. [10] replaced bone pieces with stronger titanium plate, and further strengthened it with pedicled septal flap. Rathore et al. [8] tried multiple combinations including cartilage + fascia + glue and bone + titanium plate + glue to achieve sufficient strength. Saito et al. [19] used septal cartilage, pedicled vascularized nasoseptal flap, and sealed them up with oxidized cellulose and fibrin glue. A total of seven patients underwent cranial base repairment with ‘hard + soft material’, none of them experienced CSF leak or encephalocele recurrence both during hospital stay or in long-term follow-up.

### Perioperative and postoperative complications

Among the 36 included patients, 28 patients underwent surgical treatment. Two patients died after surgery (7.14%), resulted either from fulminant pneumonia or meningitis. A total of 12 patients had at least one perioperative complication (42.86%), 7 patients underwent an uneventful recovery period (25.00%), while the perioperative course was not described in the rest 7 patients. Postoperative pituitary insufficiency, especially diabetes insipidus, was the most common perioperative complication which occurred in 6 surgery patients (21.43%). 2 patients (7.14%) developed CSF leak and meningitis (7.14%). 2 patients had noticeable peripheral nerve injury (facial nerve, abducens nerve and trigeminal nerve) after surgery (7.14%). Other complications like pneumonia, hyponatremia and extradural hematoma were also reported. 7 out of 16 patients with trans-nasal approach developed perioperative complications (43.75%); 3 out of 8 trans-palatal approached patients developed perioperative complications (37.5%); 1 out of 2 transcranial approach patient develop perioperative complications (50.00%) and 1 out of 2 combined approach patient developed complications (50.00%).

In terms of long-term treatment outcome, 3 out of 28 surgery patients experienced at least one relapse of the encephalocele which required reoperation (10.71%), most of the patients had good long-term treatment effect with improvement of symptoms before surgery. 7 patients reported to have significantly improved pituitary function (25.00%), only 1 surgery patients required hormone replacement treatment during long-term follow-up (3.57%).

## Discussions

Encephalocele can manifest through various origins, including congenital, iatrogenic, posttraumatic, or spontaneous occurrences [3]. The primary encephaloceles are linked to abnormal neural tube closure and structural fragility at bone junctions, resulting in the formation of skeletal clefts and sac-like protrusions of nervous tissue known as encephaloceles [20]. Although the precise etiology of craniofacial encephaloceles remains inconclusive, recent evidence suggests that disrupted mesenchymal migration during early fetal development leads to dysplasia of skeletal features, culminating in cleft formation and the possibility of neural herniation [20]. Trans-sphenoidal encephalocele is a relatively uncommon subtype of basal encephalocele while trans-sellar type (TSTSE), is more uncommon than classical trans-sphenoidal type, it distinguishes itself from classical trans-sphenoidal encephalocele by traversing through the greater wing of the sphenoid bone, rather than passing through the body of the sphenoid bone [15].

It is noted that the male predominance observed in TSTSE is notably different from other types of encephalocele, which typically exhibit a female predominance. This implies that the gender distribution of TSTSE differs from that of other forms of encephalocele, where females are more commonly affected [21, 22]. The potential factor underlying this phenomenon requires further investigation.

Common clinical presentations include dyspnea, visual deficits, pituitary insufficiency, nasal obstruction, recurrent meningitis and CSF leak. Most of the symptoms of TSTSE patients are either resulted from ‘mass effect’ or the structural distortion of cranial base. Patients with big encephalocele are relatively easier to detect and diagnose since the mass will obstruct the choana, thus leading to fetal dyspnea and difficult feeding, which is easily noticed by physician or parents in the first several days after birth. In addition, most of the patients presented with at least one congenital anomaly like cleft lip/palate, hypertelorism, eye problems, genital abnormalities or lingual midline mass. It is postulated that the development of congenital cleft palate/lip, hypertelorism, and eye problems may originate from the same embryonic tissues involved in the formation of the pituitary gland and other structures associated with TSTSE [5]. On the other hand, the presence of genital abnormalities is likely attributed to hypogonadism, a consequence of the encephalocele. The congenital anomalies of the patients also aid in early diagnosis of such pathology. However, in cases lacking characteristic facial features, the diagnosis of TSTSE may experience delays until adolescence or adulthood, particularly when the encephalocele is not substantial enough to cause evident obstructive mass effects [8].

To confirm the diagnosis of TSTSE and to delineate the presence of any neural or vascular components within the sac, advanced imaging studies are essential. These imaging modalities play a crucial role in providing a comprehensive assessment of the condition, aiding in accurate diagnosis, and guiding appropriate management strategies. CT scan head can help in identifying the bony defect in the skull base and the MRI scan remains the gold standard for TSTSE diagnosis since it can provide superior visualization and detailed information regarding the extent of the encephalocele as well as its contents [3]. MRI enables precise characterization of the anatomical structures involved, allowing for accurate diagnosis, treatment planning, and assessment of associated complications [13]. Endocrine evaluation plays a vital role in the management of patients with trans-sphenoidal encephaloceles. It is crucial for perioperative hormone management, optimizing postoperative recovery, and formulating long-term substitution strategies to support the patient’s growth and development [10]. By assessing the endocrine function, comprehensive hormonal profiles can be obtained, enabling the identification of any hormonal deficiencies or imbalances associated with the encephalocele. This evaluation helps guide appropriate perioperative interventions and facilitates the implementation of long-term hormone replacement therapy when necessary, ensuring optimal outcomes and addressing the patient’s overall well-being [5].

A total of 2 deaths were reported, one died from fulminant pneumonia and the other died from fulminant meningitis, both patients experienced CSF leak during perioperative recovery which can be partly attributed to the method chosen for cranial base repairment. Case 2 was given ‘hard material combination’ while case 26 was given ‘soft material combination’. In recent years, the ‘hard + soft’ sandwich technique has been extensively applied and has demonstrated good utility and strength in cranial base repairmen [23], applying simple soft flap or hard bony material may not have sufficient strength.

The surgical intervention is a double-edged sword; it can lead to severe perioperative complications and potentially lead to death, while it can also significantly improve the patient’s symptoms and improve the long-term outcome. In 28 patients who underwent surgery, 2 patients died, 3 patients required at least one reoperation due to relapse, and 1 patient needed prolonged hormone replacement therapy. The rest of the patients experienced significant improvement of the symptoms and the hormone insufficiency was also improved. For conservative treatment patient, only two patients’ long-term follow-up results were reported, neither of them had improvement of symptoms, their dyspnea and hormone insufficiency persisted after years of follow-up, which lead to growth retardation, thus influencing the patient’s quality of life. Whether to choose surgical intervention or conservative management is somehow difficult to decide due to the potential risk of each option.

In terms of approach selection, trans-nasal has been more popular in recent years since its less traumatic than transcranial approach. When compared with trans-palatal approach, trans-nasal approach also has its advantage since it does not need to split the palate, this advantage is more prominent in patients with cleft palate who are planning to have a reconstruction surgery. However, when concomitant reconstructive surgery is immediately available after the encephalocele reduction, trans-palatal approach can somehow be more desired than trans-nasal approach [8]. Simple trans-palatal approach also demonstrated less perioperative complications than trans-nasal or transcranial approach, but due to the small sample size, this conclusion should be carefully interpreted and should be further investigated.

**Fig. 4:**
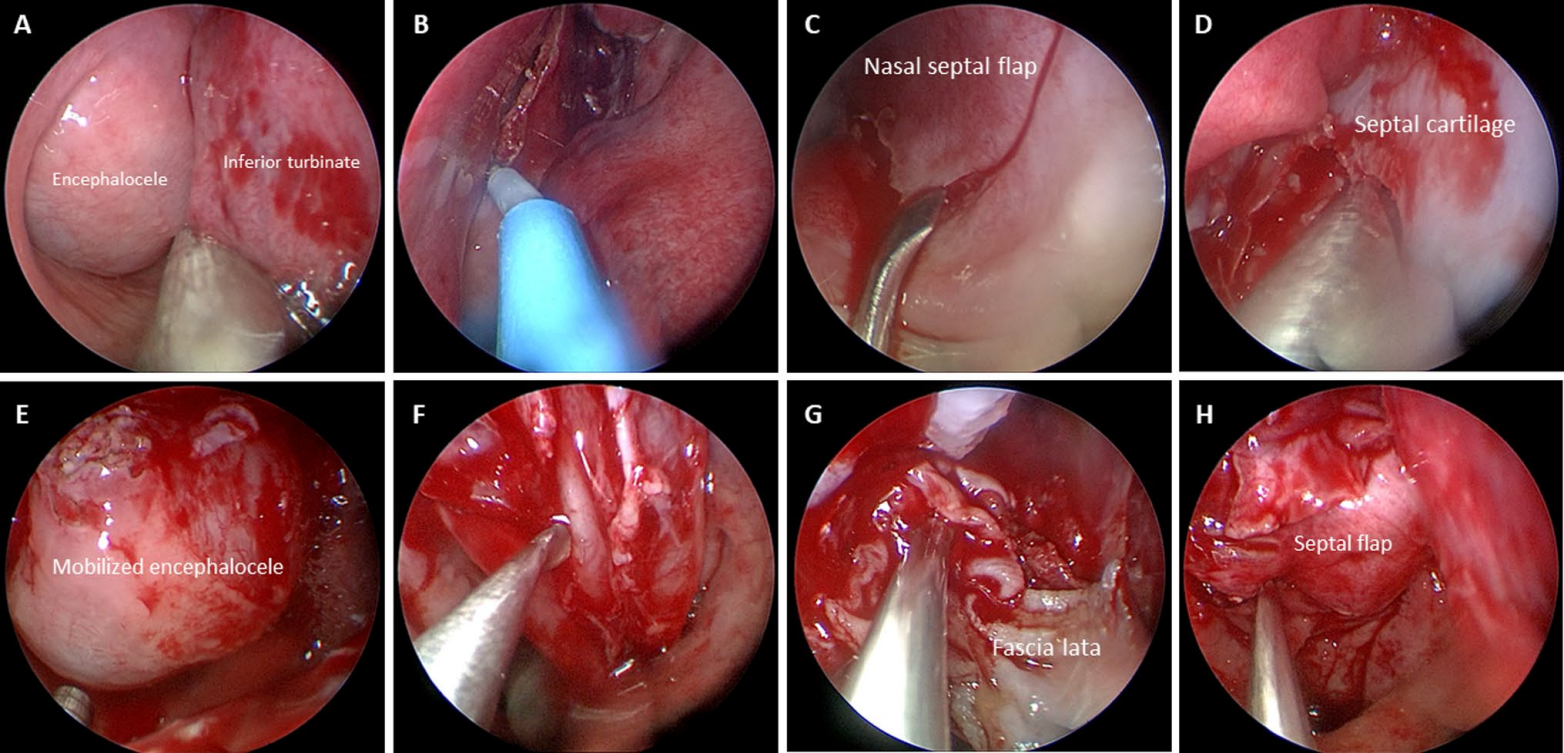
Detailed surgical procedure of our patient

We also encountered a complex 5-year-old case involving a trans-sellar encephalocele, as shown in Fig. 2b. The patient had severe obstructive sleep apnea, along with a mild cleft lip and a midline tongue mass. CT and MRI scans clearly revealed the extent of the encephalocele, which extended from the sella all the way down to the nasopharynx (Fig. 3a–d). We devised and carried out surgery for this case. During the procedure, we found that the mass was tightly adhered to the nasal septum (Fig. 4a). We attempted to separate the mass from the nasal septum while also creating a nasal septal flap (Fig. 4b–d). This revealed a round-shaped bulging mass in the nasopharynx, just in front of the sphenoid sinuses (Fig. 4e). To reduce the size of the mass, we partially removed its anterior portion (Fig. 4f) and then relocated the remaining mass back to the sella while using muscular and latissimus dorsi flap to repair the defect (Fig. 4g). Nasal septal flap was then used to further enhance the repairment (Fig. 4h).

**Fig. 5:**
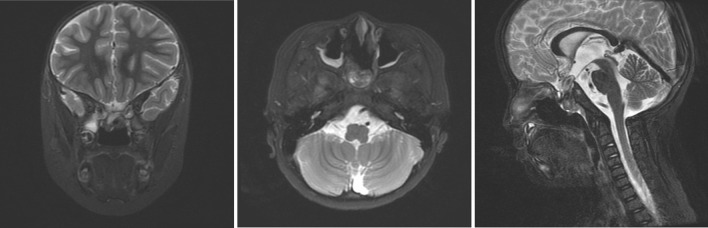
Postoperative MRI scan of the patient on 14th postoperative day. The MRI showed no cerebral edema, the encephalocele was largely reduced with only minor remnant tissue locating on the roof of the nasal cavity

After the surgery, the patient had a brief fever episode that we managed successfully with antibiotics. Fortunately, there were no complications such as CSF leakage or other issues commonly seen in similar cases. The patient also did not show signs of insipidus or hypopituitarism. Their breathing improved significantly, and they were able to breathe through the nose. The postoperative MRI revealed a diminished encephalocele with no observable signs of cerebral edema or other notable conditions (Fig. 5).

Based on our experience, precise identification of the distorted pituitary gland is pivotal for preventing postoperative pituitary crisis or diabetes insipidus. The most effective method involves locating the pituitary artery on preoperative MRI scans as well as avoiding excessive excision of the encephalocele tissue.

In conclusion, TSTSE is a disease with male predominance and is characterized by dyspnea, visual deficits, pituitary insufficiency, and other cranial base-related symptoms. Early diagnosis is crucial, and advanced imaging, particularly CT and MRI, plays a vital role in accurate assessment and management. Endocrine evaluation is essential for perioperative hormone management and long-term care. Surgical intervention can lead to significant symptom improvement but carries risks, as evidenced by reported deaths and complications. The choice between surgical intervention and conservative management should be carefully considered. Trans-nasal approach is favored for reduced trauma, but further research is needed to validate the conclusions about approach selection.

## Data Availability

All the available data are included in the article.
